# Metabolomics of mothers of children with autism, idiopathic developmental delay, and Down syndrome

**DOI:** 10.1038/s41598-024-83587-x

**Published:** 2024-12-30

**Authors:** Mariana Parenti, Shannon Shoff, Jennie Sotelo-Orozco, Irva Hertz-Picciotto, Carolyn M. Slupsky

**Affiliations:** 1 Department of Nutrition, Department of Food Science and Technology, University of California, One Shields Avenue, Davis, CA 95616 USA; 2 Department of Public Health Sciences, University of California, Davis, CA USA; 3 MIND Institute, University of California, Davis, Sacramento, CA USA; 4 Department of Food Science and Technology, University of California, Davis, CA USA; 5 Present Address: Center for Developmental Biology and Regenerative Medicine, Seattle Children’s Research Institute, Seattle, WA USA; 6 Present Address: Department of Food Science & Nutrition, Cal Poly, 1 Grand Ave San Luis Obispo, San Luis Obispo, CA 93407 USA

**Keywords:** Metabolomics, Predictive markers

## Abstract

Developmental delays have been associated with metabolic disturbances in children. Previous research in the childhood autism risk from genetics and the environment (CHARGE) case–control study identified neurodevelopment-related plasma metabolites in children, suggesting disturbances in the energy-related tricarboxylic acid (TCA) cycle and 1-carbon metabolism (1CM). Here, we investigated associations between children’s neurodevelopmental outcomes and their mothers’ plasma metabolite profiles in a subset of mother–child dyads from CHARGE, including those with autism spectrum disorder (ASD, *n* = 209), Down syndrome (DS, *n* = 76), idiopathic developmental delay (iDD, *n* = 64), and typically developed (TD, *n* = 185) controls. Multiple linear regression revealed associations between child neurodevelopmental outcomes and maternal plasma metabolites related to the TCA cycle, 1CM, and lipid metabolism. Despite profound metabolic disturbances in children with DS reported previously, few of these differences were observed in the mothers, which might reflect differences in gene dosage between children with DS and their euploid mothers. Notably differences in maternal metabolism related to ASD and iDD followed similar patterns of disturbance in previously reported metabolic signatures in children but were generally smaller in magnitude. Similar patterns of metabolic disturbances observed in mothers and their children with ASD or iDD could reflect shared genetic, mitochondrial, and/or environmental risk factors.

## Introduction

In the United States, it is estimated that approximately 1 in 6 children aged 3–17 years old have one or more developmental delays^1^. Developmental delays are lifelong conditions affecting behavior, language, and learning, which may require additional services to meet the child’s behavioral and developmental needs^2^. Furthermore, developmental delays have been associated with metabolic disturbances in children. For instance, both Down syndrome (DS) and autism spectrum disorder (ASD) have been associated with disruptions in 1-carbon metabolism (1CM) and mitochondrial dysfunction^3–8^.

In the childhood autism risks from genetics and environment (CHARGE) case–control study, an exploratory investigation showed that children with ASD were more likely to have mitochondrial dysfunction than typically developing (TD) children^9^. More recently, an analysis of plasma samples collected in the CHARGE cohort revealed that metabolic profiles differed among children with developmental disabilities. DS and ASD were particularly associated with elevated 1CM intermediates and the energy-related tricarboxylic acid (TCA) metabolites^4^. ASD, DS, and idiopathic developmental delay (iDD) were all associated with signs of peripheral mitochondrial dysfunction and altered energy metabolism^4^. Since mitochondrial function was implicated in our previous study of children in the CHARGE study and children inherit their mitochondria from their mothers, we set out to investigate whether the maternal metabolic profile was associated with the offspring’s neurodevelopment. Herein, the relationship between a child’s developmental delay and their mother’s plasma metabolic profile measured using proton nuclear magnetic resonance (^1^H-NMR) spectroscopy was investigated to identify disturbances in maternal metabolites and metabolic pathways associated with developmental outcomes in their offspring. We hypothesized that we would observe differences similar to those observed in their children.

## Methods

### Study participants

The study population included a subset of mothers of children enrolled in the childhood autism risks from genetics and environment (CHARGE) study^10^. The CHARGE study is a case–control study of children selected from three groups: children with ASD, children with developmental delay, and children from the general population as controls. Children with ASD or developmental delay were identified from the California Department of Developmental Services lists of persons receiving services for those conditions. Families have been enrolled beginning in 2003 and ongoing in 2024. Enrolled children met the following eligibility criteria: aged between 24 and 60 months, living with at least one biological parent, having one parent who speaks English or Spanish, born in California, and living in a catchment area in a specified list of California Regional Centers. The CHARGE study was carried out in accordance with all applicable guidelines and regulations for human participants and was approved by institutional review boards for the State of California Department of Developmental Services and the University of California, Davis and Los Angeles. Written informed consent was obtained before participation and any data collection. Children in the CHARGE study were clinically assessed at the UC Davis Medical Investigations of Neurodevelopmental Disorders (MIND) Institute to confirm diagnostic group. The developmental delay group was further split into children with Down syndrome (DS, based on parental report) and children with idiopathic developmental delay (iDD).

This analysis follows a previous report on metabolic differences among children in the CHARGE study by diagnostic group^4^. In this analysis, available plasma samples from mothers of these children were selected and included additional samples in the developmental delay groups to improve statistical power. Mothers of children with other genetic disorders (*n* = 11) and children with co-occurring ASD and DS (*n* = 2) were excluded. With 29 metabolites in this analysis, we selected $\alpha$ = 0.0017 for our analysis of power to detect differences in metabolite concentrations between TD and other groups. In this analysis, there were 185 mothers in the TD group, 209 mothers in the ASD group, 64 mothers in the iDD group, and 76 mothers in the DS group. With 80% power ($\beta$ = 0.2) and $\alpha$ = 0.0017, this analysis was sensitive enough to detect a difference in metabolite concentrations of at least 0.38 standard deviations (SD) between the TD and ASD groups, 0.55 SD between the TD and iDD group, and 0.52 SD between the TD and DS groups.

### Plasma metabolome analysis

Whole blood was collected in tubes containing acid citrate dextrose from each mother at the time of their child’s enrollment, processed, and the resulting plasma stored at − 80 °C until extraction. Samples were prepared for metabolite extraction and ^1^H-NMR spectra were captured as previously described^4^. Briefly, samples were thawed on ice and filtered using Amicon Ultra-0.5 mL 3000 MW centrifugal filters (Millipore, Burlington, MA). The resulting filtrate was collected, and its volume adjusted to 207 µL with ultra-pure water if insufficient sample was collected. An internal standard (Chenomx, Edmonton, AB, Canada), containing 5.0 mM 3-(trimethylsilyl)-1-propanesulfonic acid-d6 (DSS-d6), 0.2% NaN_3_, and 99.8% D_2_O was added. Each sample’s pH was adjusted to 6.8 ± 0.1 and 180 $\mu$L was loaded into a 3 mm NMR tube (Bruker, Billerica, MA). Samples were stored at 4 °C until spectral acquisition within the same day.

Spectra were acquired using the noesypr1d pulse sequence on a Bruker Avance 600 MHz spectrometer (Bruker, Billerica, MA) as previously described^4^. Spectra were manually phase- and baseline-corrected using Chenomx NMR Processor (Chenomx, Edmonton, AB, Canada, v. 8.6) and metabolite concentrations were quantified using Chenomx Profiler (Chenomx, Edmonton, AB, Canada, v. 8.6). The internal standard (DSS-d6) was used to determine each metabolite’s concentration using a compound library, which allowed for the identification and absolute quantification of compounds within a spectrum^11^. All concentrations were corrected for dilution by multiplying the final concentrations by the ratio of total volume to sample volume.

### Covariate selection

**Fig. 1:**
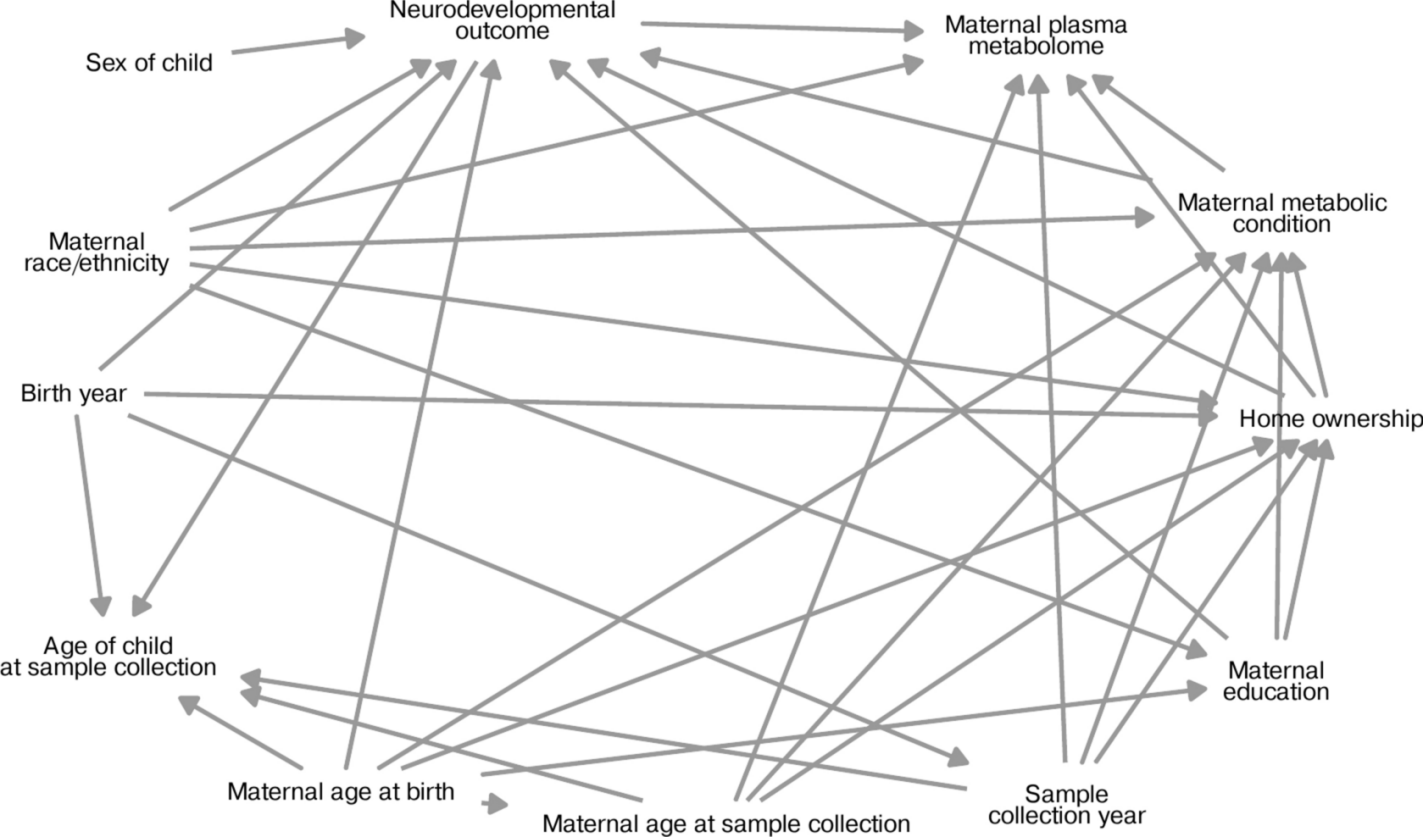
Directed acyclic graph (DAG) to identify a sufficient set of control variables for the analysis of the total association between the child’s diagnosis and the maternal plasma metabolome, which included maternal age at sample collection, child’s sex, maternal metabolic condition, maternal race/ethnicity, home ownership, and year of sample collection.

This study aimed to estimate the effect of the child’s neurodevelopmental diagnosis (ASD, iDD, or DS) compared to TD controls on the maternal plasma metabolome. We encoded our assumptions using a directed acyclic graph (DAG), which was used to select a minimal sufficient adjustment set to estimate this effect using the R package *dagitty* (v. 0.3-1)^12^. The DAG included maternal age at the child’s birth, maternal age at sample collection, year of birth, year of collection, the child’s age at collection, maternal educational attainment, maternal race/ethnicity, home ownership status, the child’s sex, maternal metabolic condition, and the child’s diagnosis (Fig. 1). We used the DAG to assist in identification of a sufficient set of control variables for analysis of the total association between the child’s neurodevelopmental diagnosis and the maternal plasma metabolome, which included maternal age at sample collection, child’s sex, maternal metabolic condition, maternal race/ethnicity, home ownership, and year of sample collection.

### Statistical analysis

Differences in the study population between TD controls and ASD, iDD, and DS groups were assessed using χ^2^ tests for categorical variables and Kruskal–Wallis tests for continuous variables. Pairwise group comparisons for variables that significantly differed were conducted using pairwise proportion tests corrected for multiple comparisons using the Bonferroni–Holm correction and Dunn’s test, respectively. Plasma metabolites were corrected for dilution and log_10_-transformed to improve skewed distributions. To test our hypothesis that the metabolic changes in maternal plasma associated with child developmental outcome would be reflective of the changes observed in child’s plasma, we selected and analyzed the following 29 metabolites. First, we included the metabolites we previously observed to be associated with at least one developmental outcome. These metabolites were 2-aminobutyrate, 2-hydroxybutyrate, 2-oxoglutarate, 3-hydroxyisobutyrate, acetate, acetoacetate, alanine, arginine, asparagine, betaine, carnitine, choline, *cis*-aconitate, creatinine, dimethyl sulfone, glutamate, glycine, histidine, lactate, lysine, *N,N*-dimethylglycine, *O*-acetylcarnitine, ornithine, serine, succinate, and urea. We further included the TCA cycle intermediates fumarate and pyruvate, as well as lipid-related metabolites 3-hydroxybutyrate and acetone. Though these metabolites were not noted to differ across diagnostic groups in the children’s plasma samples, other metabolite changes were noted in these pathways. Thus, these metabolites were included to provide a more complete picture of these metabolic pathways in maternal plasma. Metabolite concentrations are presented as μmol/L in Supplementary Table 1.

The association between neurodevelopmental outcome and each individual plasma metabolite was assessed using multiple linear regression (MLR) adjusted for covariates and FDR-corrected using the Benjamini–Hochberg procedure^13^. We considered FDR-corrected *q* < 0.05 to be statistically significant and FDR *q* < 0.1 to represent statistical trends. We evaluated the effect size for comparisons between the TD reference group and ASD, iDD, and DS using Cliff’s delta ($\delta$) in the *effsize* package (v. 0.8.1)^14^. In this analysis, |$\delta$|< 0.33 was considered a small effect, 0.33 ≤|$\delta$|< 0.474 was considered a medium effect, and |δ|≥ 0.475 was considered a large effect. All analyses were conducted using the R statistical language (v 4.2.2) in RStudio^15^.

## Results

Characteristics of mothers and their children in the study population are presented in Table 1. While TD children were frequency-matched by sex and age to ASD cases in the CHARGE study, the sex distribution was not matched among the cases with iDD or DS. The children’s ages at sample collection did not differ between diagnostic groups. Mothers in the DS group were older compared to all other groups at their child’s birth and at sample collection, while mothers in the iDD group were younger than TD mothers at their child’s birth. Participants in this sample were recruited between 2003 and 2016. Children in the DS group recruited to CHARGE were born on average in later years and children with ASD recruited to CHARGE were more commonly born in the earlier years. Thus, blood samples were collected later from mothers in the DS group compared to the blood samples collected from the TD group and earlier from mothers in the ASD group compared to all other groups. Children with iDD were more likely to identify as Hispanic. Mothers in the iDD group were more likely to identify as Black or Hispanic and less likely to identify as white. In the global χ^2^ test, maternal education differed by neurodevelopmental diagnosis, though the proportion of mothers at each education level did not differ by diagnosis. However, when we investigated maternal education level as a dichotomous variable (some college or less, bachelor’s degree or higher), mothers in the TD and ASD groups were more likely to have a bachelor’s degree or higher compared to mothers in the iDD group. Additionally, mothers in the iDD group were less likely to have attained a bachelor’s degree or higher. Mothers of TD children were more likely to own their homes relative to mothers in the iDD group. The global χ^2^ test revealed a difference in maternal metabolic condition by neurodevelopmental diagnosis, but the proportion of mothers at each level of maternal metabolic condition did not differ by diagnosis. However, when we compared the mothers in the ASD group to mothers of children without ASD, we found that mothers in the ASD group were less likely to be overweight (25 ≤ BMI < 30) without any metabolic conditions and were more likely to have hypertensive disorders compared to mothers of children without ASD.

**Table 1:** Participant characteristics by neurodevelopmental diagnosis: typically developing controls (TD), autism spectrum disorder (ASD), idiopathic developmental delay (iDD), and Down syndrome (DS).

	TD (*n* = 185)	ASD (*n* = 209)	iDD (*n* = 64)	DS (*n* = 76)	*p*
Mother’s age at sample collection, years	35 (32, 38)^a^	34 (31, 38)^a^	32 (28, 37)^a^	39 (34, 43)^b^	< 0.001
Child’s age at sample collection, months	44 (35, 53)	45 (36, 53)	45 (41, 53)	47 (42, 52)	0.313
Missing	0	7	1	15	
Collection year	2008 (2006, 2011)^b^	2007 (2005, 2009)^a^	2009 (2007, 2011)^bc^	2010 (2007, 2012)^c^	< 0.001
Mother’s age at birth, years	31 (28, 34)^b^	31 (27, 35)^ab^	28 (24, 32)^a^	36 (31, 39)^c^	< 0.001
Birth year	2004 (2003, 2007)^b^	2004 (2001, 2006)^a^	2005 (2003, 2006)^bc^	2006 (2004, 2009)^c^	< 0.001
Child’s sex					
Male	137 (74.1%)^ab^	157 (75.1%)^a^	37 (57.8%)^bc^	40 (52.6%)^c^	< 0.001
Female	48 (25.9%)^ab^	52 (24.9%)^a^	27 (42.2%)^bc^	36 (47.4%)^c^	
Child’s race/ethnicity					
White	95 (51.9%)	98 (46.9%)	22 (34.4%)	33 (44.6%)	0.002
Black	5 (2.7%)	6 (2.9%)	7 (10.9%)	3 (4.1%)	
Asian	6 (3.3%)	12 (5.7%)	1 (1.6%)	2 (2.7%)	
Hispanic, any race	46 (25.1%)^a^	72 (34.4%) ^ab^	30 (46.9%) ^b^	30 (40.5%)^ab^	
Other or multi-racial	31 (16.9%)	21 (10.0%)	4 (6.2%)	6 (8.1%)	
Missing	2	0	0	2	
Mother’s race/ethnicity					
White	125 (67.6%)^a^	121 (57.9%)^ab^	27 (42.2%)^b^	42 (55.3%)^ab^	< 0.001
Black	5 (2.7%)^a^	9 (4.3%) ^a^	10 (15.6%)^b^	4 (5.3%)^ab^	
Asian	16 (8.6%)	20 (9.6%)	3 (4.7%)	3 (3.9%)	
Hispanic, any race	33 (17.8%)^a^	53 (25.4%)^ab^	23 (35.9%)^b^	25 (32.9%)^ab^	
Other or multi-racial	6 (3.2%)	6 (2.9%)	1 (1.6%)	2 (2.6%)	
Mother’s education					
High school/GED or less	28 (15.1%)	31 (14.8%)	17 (26.6%)	13 (17.1%)	0.041
Some college	54 (29.2%)	78 (37.3%)	29 (45.3%)	30 (39.5%)	
Bachelor degree	73 (39.5%)	73 (34.9%)	15 (23.4%)	24 (31.6%)	
Graduate or professional degree	30 (16.2%)	27 (12.9%)	3 (4.7%)	9 (11.8%)	
Parental home ownership					
No	44 (23.8%) ^a^	74 (35.4%) ^ab^	27 (42.2%) ^b^	29 (38.2%) ^ab^	0.012
Yes	141 (76.2%) ^a^	135 (64.6%) ^ab^	37 (57.8%) ^b^	47 (61.8%) ^ab^	
Maternal metabolic condition					
BMI < 25, no metabolic conditions	89 (48.1%)	106 (50.7%)	24 (37.5%)	35 (46.1%)	0.014
25 ≤ BMI < 30, no metabolic conditions	47 (25.4%)	30 (14.4%)	16 (25.0%)	20 (26.3%)	
BMI ≥ 30, no metabolic conditions	29 (15.7%)	33 (15.8%)	12 (18.8%)	11 (14.5%)	
Any hypertensive disorder without diabetes, at any BMI	10 (5.4%)	23 (11.0%)	2 (3.1%)	2 (2.6%)	
Any diabetes, at any BMI	10 (5.4%)	17 (8.1%)	10 (15.6%)	8 (10.5%)	

Categorical variables are presented as n (%) and associations with neurodevelopmental diagnosis were tested using χ^2^ tests for categorical variables.

Continuous variables are presented as median (IQR) and associations with neurodevelopmental diagnosis were tested using Kruskal–Wallis test.

Within each row, groups that do not share the same letter were statistically different in pairwise proportion tests adjusted for multiple comparisons.

### Association between maternal plasma metabolites and child’s diagnosis

Multiple linear regressions were used to investigate differences in the maternal plasma metabolic profile in ASD, iDD, or DS groups relative to the TD group after adjustment for covariates (Table 2). All associations that were significant before FDR correction had small $\delta$ values, ranging from − 0.25 to 0.26. Relative to mothers in the TD group, mothers in the ASD group had higher plasma concentrations of lactate ($\beta$ = 0.076, *p* = 0.001, *q* = 0.020) and alanine ($\beta$ = 0.022, *p* = 0.041, *q* = 0.240), as well as lower plasma concentrations of 3-hydroxybutyrate ($\beta$ = − 0.134, *p* = 0.002, *q* = 0.029), acetate ($\beta$ = − 0.064, *p* = 0.011, *q* = 0.077), and acetone ($\beta$ = − 0.043, *p* = 0.011, *q* = 0.077).

**Table 2:** Multiple linear regression (MLR) analysis of log_10_-transformed plasma metabolite concentrations for mothers of children with autism spectrum disorder (ASD; *n* = 209), idiopathic developmental delay (iDD; *n* = 64), and Down syndrome (DS; *n* = 76), each compared to mothers of children with typical development (TD; *n* = 185).

Metabolite	Median concentration (IQR), μmol/L	Diagnosis	Coefficient	95% CI	*p*	*q*	δ
2-Aminobutyrate	18.3 (14.7–22.6)	ASD	− 0.024	(− 0.051, 0.004)	0.097	0.334	− 0.12
		DS	− 0.003	(− 0.042, 0.036)	0.861	0.984	0.02
		iDD	0.004	(− 0.037, 0.044)	0.861	0.892	0.12
2-Hydroxybutyrate	16.8 (11.9–23.5)	ASD	− 0.042	(− 0.085, 0.001)	0.058	0.278	− 0.12
		DS	− 0.047	(− 0.107, 0.014)	0.134	0.723	− 0.10
		iDD	0.025	(− 0.038, 0.088)	0.435	0.719	0.10
2-Oxoglutarate	17.2 (11.6–28.4)	ASD	0.035	(− 0.018, 0.088)	0.193	0.432	− 0.01
		DS	0.025	(− 0.049, 0.098)	0.514	0.747	0.13
		iDD	0.025	(− 0.051, 0.102)	0.521	0.796	0.12
**3-Hydroxybutyrate**	19.7 (10.8–50.8)	**ASD**	− **0.134**	**(**− **0.219, −0.050)**	**0.002**	**0.029**	− **0.18**
		DS	− 0.063	(− 0.182, 0.056)	0.302	0.747	− 0.09
		iDD	0.013	(− 0.110, 0.137)	0.835	0.892	0.01
3-Hydroxyisobutyrate	8.3 (6.6–10.4)	ASD	− 0.016	(− 0.045, 0.013)	0.276	0.466	− 0.06
		DS	− 0.043	(− 0.084, − 0.003)	0.037	0.356	− 0.17
		iDD	− 0.016	(−0.059, 0.026)	0.446	0.719	− 0.02
**Acetate**	14.4 (11.1–20.1)	**ASD**	− **0.064**	**(**− **0.112,** − **0.015)**	**0.011**	**0.077**	− **0.08**
		DS	0.002	(− 0.067, 0.070)	0.963	0.992	− 0.05
		iDD	− 0.087	(− 0.157, − 0.016)	0.017	0.221	− 0.25
Acetoacetate	1.4 (0.8–3)	ASD	− 0.017	(− 0.075, 0.041)	0.564	0.681	0.05
		DS	− 0.004	(− 0.086, 0.077)	0.916	0.984	− 0.06
		iDD	0.065	(− 0.020, 0.149)	0.133	0.552	0.15
**Acetone**	39.4 (31.6–51.1)	**ASD**	**−0.043**	**(**− **0.075,** − **0.010)**	**0.011**	**0.077**	− **0.17**
		DS	− 0.006	(− 0.052, 0.039)	0.789	0.984	− 0.01
		iDD	0.024	(− 0.023, 0.072)	0.318	0.711	0.06
Alanine	336.1 (284.1–402.7)	ASD	0.022	(0.001, 0.043)	0.041	0.240	0.06
		DS	0.021	(− 0.009, 0.050)	0.174	0.723	0.17
		iDD	0.013	(− 0.018, 0.044)	0.409	0.719	0.09
Arginine	35.4 (26.9–45)	ASD	− 0.026	(− 0.058, 0.006)	0.115	0.334	0.00
		DS	0.003	(-0.042, 0.048)	0.903	0.984	− 0.06
		iDD	− 0.031	(− 0.078, 0.016)	0.196	0.632	− 0.09
Asparagine	38.5 (32.9–44.4)	ASD	0.014	(− 0.014, 0.042)	0.317	0.466	− 0.04
		DS	0.015	(− 0.024, 0.054)	0.451	0.747	0.07
		iDD	0.001	(− 0.040, 0.041)	0.974	0.974	0.02
Carnitine	24.8 (16.9–31.5)	ASD	0.019	(− 0.018, 0.055)	0.313	0.466	− 0.07
		DS	− 0.015	(− 0.067, 0.036)	0.555	0.767	0.09
		iDD	0.048	(− 0.006, 0.101)	0.080	0.389	0.12
Choline	15 (11.4–19.4)	ASD	0.015	(− 0.015, 0.046)	0.322	0.466	− 0.02
		DS	− 0.003	(− 0.046, 0.039)	0.873	0.984	0.07
		iDD	0.052	( 0.007, 0.096)	0.023	0.221	0.17
*cis*-Aconitate	67.2 (40.4–109.4)	ASD	0.036	(− 0.020, 0.092)	0.209	0.432	0.00
		DS	0.000	(− 0.079, 0.078)	0.992	0.992	0.10
		iDD	0.116	(0.035, 0.197)	0.005	0.154	0.24
Creatinine	49.7 (44.3–56.2)	ASD	− 0.004	(− 0.020, 0.011)	0.593	0.688	− 0.10
		DS	− 0.010	(− 0.032, 0.012)	0.391	0.747	0.02
		iDD	− 0.012	(− 0.035, 0.011)	0.308	0.711	− 0.06
Dimethyl sulfone	4.7 (3.5–6.3)	ASD	− 0.027	(− 0.078, 0.023)	0.292	0.466	− 0.17
		DS	0.029	(− 0.042, 0.100)	0.427	0.747	0.05
		iDD	0.041	(− 0.033, 0.115)	0.278	0.711	− 0.02
Fumarate	2.1 (1.8–2.6)	ASD	0.016	(− 0.007, 0.038)	0.170	0.432	0.07
		DS	0.022	(− 0.009, 0.054)	0.170	0.723	0.11
		iDD	0.023	(− 0.010, 0.056)	0.175	0.632	0.09
Glutamate	137.3 (84.8–230.2)	ASD	− 0.035	(− 0.088, 0.018)	0.195	0.432	− 0.12
		DS	− 0.030	(− 0.104, 0.044)	0.423	0.747	− 0.02
		iDD	− 0.017	(− 0.094, 0.060)	0.663	0.801	0.00
Glycine	216.8 (174.6–278.8)	ASD	0.010	(− 0.019, 0.039)	0.503	0.635	0.00
		DS	0.029	(− 0.012, 0.069)	0.162	0.723	0.21
		iDD	0.017	(− 0.025, 0.059)	0.439	0.719	0.06
Histidine	65.5 (57.4–74.1)	ASD	− 0.002	(− 0.022, 0.017)	0.806	0.835	− 0.03
		DS	− 0.009	(− 0.037, 0.018)	0.515	0.747	− 0.05
		iDD	− 0.007	(− 0.036, 0.021)	0.621	0.801	0.05
**Lactate**	3289.7 (2591.9–4233)	**ASD**	**0.076**	**(0.032, 0.120)**	**0.001**	**0.020**	**0.12**
		DS	0.069	(0.007, 0.130)	0.029	0.356	0.24
		iDD	0.070	(0.006, 0.134)	0.033	0.241	0.17
Lysine	141.4 (119.8–162)	ASD	0.004	(− 0.016, 0.024)	0.667	0.726	0.00
		DS	0.010	(− 0.018, 0.038)	0.476	0.747	0.13
		iDD	0.013	(− 0.016, 0.043)	0.363	0.719	0.14
*N,N*-Dimethylglycine	1.8 (1.4–2.3)	ASD	− 0.008	(− 0.028, 0.012)	0.419	0.552	− 0.03
		DS	0.009	(− 0.019, 0.038)	0.511	0.747	0.08
		iDD	0.007	(− 0.023, 0.036)	0.655	0.801	0.09
*O*-Acetylcarnitine	4.7 (3.6–6.2)	ASD	− 0.028	(− 0.059, 0.004)	0.086	0.334	− 0.10
		DS	0.009	(− 0.035, 0.053)	0.696	0.917	0.02
		iDD	0.013	(− 0.033, 0.059)	0.575	0.801	0.06
Ornithine	101.7 (82.2–120.8)	ASD	0.012	(− 0.016, 0.041)	0.398	0.549	0.00
		DS	0.015	(− 0.025, 0.056)	0.454	0.747	0.14
		iDD	0.024	(− 0.018, 0.066)	0.259	0.711	0.08
Pyruvate	408.4 (293.8–483.8)	ASD	0.039	(− 0.024, 0.102)	0.230	0.444	− 0.03
		DS	0.034	(− 0.054, 0.122)	0.451	0.747	0.16
		iDD	− 0.009	(− 0.101, 0.083)	0.847	0.892	0.08
Serine	105.8 (88.1–123.1)	ASD	− 0.005	(− 0.026, 0.017)	0.676	0.726	−0.06
		DS	0.014	(− 0.016, 0.044)	0.356	0.747	0.14
		iDD	0.004	(-0.027, 0.035)	0.810	0.892	0.06
Succinate	17.6 (14.1–22.4)	ASD	0.025	(− 0.005, 0.055)	0.106	0.334	0.06
		DS	0.048	(0.006, 0.091)	0.026	0.356	0.26
		iDD	0.040	(− 0.004, 0.084)	0.076	0.389	0.19
Urea	1529.5 (1245.1–1845.2)	ASD	− 0.002	(− 0.027, 0.023)	0.860	0.860	− 0.07
		DS	− 0.012	(− 0.048, 0.023)	0.488	0.747	0.02
		iDD	0.009	(− 0.027, 0.046)	0.619	0.801	− 0.02

MLR were conducted on log_10_-transformed data and adjusted for maternal age at sample collection, child’s sex, maternal metabolic condition, maternal race/ethnicity, home ownership, and year of sample collection. The coefficient and 95% confidence interval (CI) are presented. Metabolites with coefficients with *q*-value < 0.10 are bolded. Raw and false discovery rate corrected *p*-values (*q*) are presented. The effect size was measured using Cliff’s delta (δ) for the comparisons between ASD and TD, DS and TD, and iDD and TD.

Relative to mothers in the TD group, mothers in the iDD group had higher plasma concentrations of *cis*-aconitate ($\beta$ = 0.116, *p* = 0.003, *q* = 0.154), choline ($\beta$ = 0.052, *p* = 0.023, *q* = 0.221), and lactate ($\beta$ = 0.070, *p* = 0.033, *q* = 0.241), as well as lower plasma concentrations of acetate ($\beta$ = − 0.087, *p* = 0.017, *q* = 0.221).

Relative to mothers in the TD group, mothers in the DS group had higher plasma concentrations of lactate ($\beta$ = 0.069, *p* = 0.029, *q* = 0.356) and succinate ($\beta$ = 0.048, *p* = 0.026, *q* = 0.356), as well as lower concentrations of 3-hydroxyisobutyrate ($\beta$ = − 0.043, *p* = 0.037, *q* = 0.356).

## Discussion

In this study, we investigated the plasma metabolic profiles of mothers of children with ASD, iDD, and DS compared to mothers of TD controls, with more subtle results. We observed that plasma 3-hydroxybutyrate and lactate concentrations significantly differed (*q* < 0.05) and acetate and acetone marginally differed (*q* < 0.1) between mothers of children with ASD compared to mothers of TD children (Table 2). We have previously examined the children’s plasma metabolic profiles^4^, identifying disturbances in 1CM, the TCA cycle, the transsulfuration pathway, and lipid-related metabolites. Notably, plasma lactate was also elevated in children in the ASD group, though the difference in maternal metabolism was smaller than differences observed between their children^4^. Elevated lactate in mothers of children in the ASD group could suggest mitochondrial dysfunction. Indeed, elevated blood lactate has been used as a clinical sign of mitochondrial respiratory chain disorders^16,17^. ASD has a multifactorial etiology, and mitochondrial dysfunction is thought to be a contributing factor^18^. Mitochondria are inherited matrilineally, so these signs of maternal mitochondrial dysfunction could contribute to ASD risk.

The mitochondria are also cellular sites of lipid metabolism. In this study, we also observed a pattern of perturbed lipid metabolism in mothers of children with ASD that was not observed in their children. In addition to lower plasma acetate concentrations (*q* < 0.1), we observed lower plasma concentrations of the ketones 3-hydroxybutyrate (*q* < 0.05) and acetone (*q* < 0.1) in mothers in the ASD group compared to mothers in the TD group. This pattern suggests that lipid metabolism may be disturbed in mothers of children with ASD. Interestingly, we have recently shown that lower concentrations of maternal serum 3-hydroxybutyrate during the third trimester of pregnancy were associated with having a child with non-typical neurodevelopment in a prospective birth cohort of younger siblings of children with ASD^19^. If the pattern of altered lipid metabolism observed here also existed during pregnancy, it is possible that reduced circulating lipid-related metabolites could lead to reduced substrates for fetal brain development.

Surprisingly, while we previously observed profound metabolic disturbances in children with DS related to lipid metabolism, energy metabolism, and 1CM^4^, few of these differences were observed in the mothers. Indeed, we observed few differences in metabolite concentrations between the mothers of children with DS and mothers of children with TD at all. The number of children with DS was substantially smaller than the number of ASD children, hence the statistical power was lower. On the other hand, previous studies have suggested the metabolic perturbations observed in DS compared to controls could be related to the gene dosage effect in individuals with trisomy 21 compared to euploid siblings^20–22^. It might be that we do not observe similar patterns of metabolic disturbances between mothers and their children with DS because the mothers are euploid. While there is some evidence of genetic risk factors for DS related to nondisjunction during meiosis^23,24^, DS is not typically considered an inherited condition. On the other hand, genetics (including mitochondrial DNA) are thought to play an important role in the development of ASD and developmental disabilities^25–27^. Likewise, environmental exposures and gene-by-environment interactions may also play a role in the etiology of ASD and developmental delays^28,29^. Thus, the handful of metabolic disturbances observed in mothers and their children with ASD may reflect shared genetic, mitochondrial, and/or environmental risk factors within a family.

Our analyses have several limitations. First, in this analysis of maternal plasma, we observed much smaller differences between mothers in the ASD, iDD, or DS groups compared to mothers in the TD group than we observed in the children’s plasma. The smaller differences in mothers that did not reach significance might have been chance findings or might represent true meaningful differences across child’s neurodevelopmental groups being smaller for the mothers as compared with their children; in the latter case, the sample size may have provided inadequate power—despite being large for a metabolomics study. Notably, fewer differences were observed for the two smaller diagnostic groups (iDD and DS), as compared with ASD. Second, while we accounted for potential confounders in this analysis with an evidence-based, causally informed set of adjustment variables, we cannot rule out residual confounding. For example, it is possible that differences in diets between mothers of TD children versus those of other diagnostic groups could contribute to the changes we see in energy- and lipid-related metabolites. For instance, ASD is associated with food selectivity and this selectivity explains the relationship between higher ASD traits and poor diet quality^30^. Furthermore, this food selectivity in children with ASD can impact the diets within their household^31^. Thus, the elevated lactate and lower 3-hydroxybutyrate we report here could be confounded by unmeasured dietary differences between groups.

Finally, this study was associational, without the ability to establish the directionality of the observed associations. This investigation relied on maternal samples collected several years post-partum, which, for our purposes, were of interest as a proxy for maternal metabolism during pregnancy. Specifically, although ASD and iDD diagnoses are made, respectively, years or months after delivery, the origins of those conditions are considered likely to occur during gestation, and DS is a genetic disorder set in motion before conception. Both ASD and iDD may involve interactions of environmental factors with genes, again pointing to some pre- and/or peri-conception causal contributions. For these reasons, we analyzed the child’s diagnosis as a predictor of maternal metabolome, while recognizing potential or plausibility for the opposite directionality. Indeed, no inference can be made from this study as to the directionality of the observed associations, nor can causality be inferred.

Nonetheless, the research presented herein demonstrated several differences in maternal plasma metabolic profiles comparing mothers whose children had an ASD diagnosis with those whose children were developing typically. The alterations, which are chiefly related to mitochondrial function, map to similar patterns previously observed in their children. We also observed indications of altered lipid metabolism in mothers of children with ASD. Further research on maternal lipid and mitochondrial metabolism and their relationship with offspring brain development is warranted. Additionally, future metabolomic research conducted in fathers and unaffected siblings would help to contextualize results reported here.

## Supplementary Information

Supplementary Information.

## Data Availability

The datasets generated and/or analyzed during the current study are available in Supplementary Table 1.
